# Topical Estrogen Treatment Augments the Vaginal Response to *Escherichia coli* Flagellin

**DOI:** 10.1038/s41598-020-64291-y

**Published:** 2020-05-21

**Authors:** Anna Stanton, Catherine Mowbray, Marcelo Lanz, Karen Brown, Paul Hilton, Alison Tyson-Capper, Robert S. Pickard, Ased S. M. Ali, Judith Hall

**Affiliations:** 1 Institute for Cell & Molecular Biosciences, Newcastle University, Newcastle upon Tyne, UK; 2 Institute of Cellular Medicine, Newcastle University, Newcastle upon Tyne, UK; 3 Newcastle upon Tyne Hospitals NHS Foundation Trust, Newcastle upon Tyne, UK; 4 Clinical Academic Office & Institute of Health & Society, Newcastle University, Newcastle upon Tyne, UK; 5 Present Address: Leica Biosystems, Newcastle upon Tyne, UK; 6 Present Address: Nippon Genetics Europe, Düren, Germany; 7 Present Address: Mid Yorkshire Hospitals, Aberford Rd, Wakefield, UK; 8 Clinical Academic Office & Institute Health & Society, Newcastle University;, Newcastle upon Tyne NHS Foundation Trust, UK

**Keywords:** Urinary tract infection, Urinary tract infection

## Abstract

The female climacteric or menopausal process characterised by reduced estrogen, associates with an increased risk of recurrent urinary tract infections (rUTIs) linked to uropathogenic *Escherichia coli* (UPEC). Clinically, topical vaginal estrogen treatment has a prophylactic effect against such infections. The aim of this study was to investigate, *in vitro*, the effects of a topical estrogen treatment on vaginal epithelial responses following challenge with *E.coli* flagellin mimicking an UPEC challenge. Immortalised vaginal epithelial cells (VK2 E6/E7), modelling the vaginal epithelium were treated with either 4 nM 17β-estradiol (E) for seven days, 50 ng/ml *E.coli* flagellin (F) for 12 h, or 4 nM 17β-estradiol plus 50 ng/ml flagellin (E + F(12 h)). RNA was analysed by microarray gene profiling using the Illumina HumanHT-12 v 4 Expression Beadchip. Following E + F treatments expression of genes encoding host defence molecules including *DEFβ4A*, *DEFB103A*, *LCN2* as well as those associated with keratinisation eg *CNFN* and *SPRR* family genes were significantly enhanced (P < 0.05) compared to either E or F treatments alone. Mutation of estrogen responsive elements (EREs) identified in the *DEFβ4* gene promoter abolished the augmented gene expression suggesting estrogen functioned directly through a regulatory mechanism involving ESR1/2. Ingenuity pathway analyses also suggested the pro-inflammatory cytokine IL-17A to regulate the vaginal host defences during infection. Pre-treating VK2 E6/E7 cells with estrogen (4 nM) and challenging with 1L-17A & F (12 h) significantly enhanced *DEFβ4, DEF103A* and *S100A7* expression (P < 0.05). Origins of vaginal IL-17 *in vivo* remain unclear, but patient biopsies support γδ T cells located within the vaginal epithelium. These data suggest that the vaginal antimicrobial response induced by flagellin activation of Toll-like Receptor 5 cell signalling is augmented following topical estrogen application.

## Introduction

Decreased circulating concentrations of the steroid hormone estrogen characterise the female climacteric, which in some women coincides with the onset of urogenital pathologies, including vaginal atrophy and rUTIs Topical vaginal estrogen treatments, but not systemic estrogen therapy have been shown to alleviate rUTIs in post-menopausal women^1,2^.

Uncomplicated rUTIs experienced by post-menopausal women are generally characterised as ascending infections that, in more than 75% of cases, link to *Esherichia coli*^3^. Such bacteria referred to as uropathogenic *E.coli* (UPEC) originate in the gut, colonise the vaginal epithelium and migrate via the urethra to the bladder where they cause symptomatic infections^4,5^. The infection process is facilitated by UPEC displaying factors such as flagella and pili, which support both motile and adherent phenotypes that underpin their virulence in the urinary tract^6^. Protection of the female urogenital tract from such uropathogens, pre-menopausally, is mediated by an array of constitutive and induced host innate defence factors as well as the vaginal microbiota. This microbiota is dominated by *Lactobacilli*^7,8^, which metabolise glycogen derived simple sugars to lactic acid, resulting in an acid micro-environment hostile to UPEC survival^9^. The post-menopausal state, in contrast, is associated with a more neutral vaginal pH, which supports UPEC persistence and colonisation, and coincides with an increased susceptibility to rUTIs^10^.

In murine models, rUTIs have also been explained by the presence of intracellular and quiescent *E.coli* communities, establishing and residing within the bladder epithelium^11^. The intracellular location of these bacterial communities means they are protected from the host defences and the effects of externally administered antimicrobials, and their persistence provides a constant bacterial reservoir able to seed new infections. While there is limited, but direct evidence to support this infection model in post-menopausal women^12^, it does not easily align to clinical data showing vaginal topical treatments are associated with a reduced incidence of UTIs^1,13^.

The standard clinical treatment for rUTIs is antibiotics, but this option is complicated by the relative ineffectiveness of multiple and prolonged courses that promote resistant strains of the main causative organism UPEC^14^. Pre-conditioning of the vaginal or urethral epithelium to hinder *E.coli* colonisation and migration represents an alternative treatment for rUTIs and one such treatment available to post-menopausal women suffering rUTIs is topical vaginal estrogen treatment^1^. Mechanisms proposed to explain how topical estrogen functions in protecting from rUTI include increased vaginal glycogen stores favouring colonisation of the epithelium by *Lactobacilli*^15^, a strengthening of the urothelial barrier and the increased antimicrobial capacity of the bladder defences^16^.

*In vitro* data also support the clinical observations that vaginal estrogen plays a key role in boosting the bladder innate defences with epithelial integrity and host effectors including the host defence peptide, human β-defensin-2(hBD2), elevated in response to topical treatments^16,17^. Analyses of vaginal biopsy material from post-menopausal women with vaginal atrophy and pro-lapse supports local estrogen therapy to impact epithelial structure and barrier function through increased expression of genes associated with cell proliferation, differentiation and defence^18^ and extracellular matrix protein synthesis^19^. However, the effects of estrogen on the vaginal host innate defences particularly during periods associated with potential UPEC infections are less well described.

While topical vaginal estrogen treatments help protect against rUTIs not all women can tolerate the therapy with vaginal bleeding or spotting, vaginal irritation, burning and itching reported as common adverse events^1^. Therefore further knowledge of the mechanisms by which the steroid hormone functions to help protect against rUTIs is necessary; this information may help underpin the development of non-hormonal alternative topical treatments that mimic the protective effects of estrogen, but carry reduced side-effects. The aim of this study was to investigate the mechanisms by which estrogen influences the innate defences of the vaginal epithelium, particularly in response to flagellin challenge utilized to model a putative UTI.

## Methods

### Patient samples

The patient study protocol exploring the effects of estrogen on the vaginal defences was carried out following a minor project amendment to REC Reference 09/H0905/15 (Newcastle upon Tyne Hospitals NHS Trust R&D Reference 4841). Postmenopausal patients undergoing either hysterectomy or vaginal prolapse surgery were recruited to the study from the Urogynaecology department of Newcastle upon Tyne Hospitals NHS Foundation Trust and provided written informed consent. Patients groups reflected those either prescribed vaginal estrogen treatment (Vagifem® [estradiol hemihydrate equivalent to estradiol] 10 mcg twice weekly (n = 41, median age of 57 years) or not (n = 53, median age of 60 years) (Table 1S). Each of the 94 patients donated vaginal fluid and tissue samples collected in accordance with relevant guidelines and regulations, and these were analysed either by ELISA or gene expression using qPCR.

**Table 1:** Microarray results of gene probes specifically encoding proteins/peptides involved in antimicrobial activity, keratinisation and inflammation ie epithelial barrier defence, and differentially expressed in relation to E + F (12/24 h) treatments.

Gene Symbol	Probe Fold Change	Protein	Function	E	F(12 h)	E + F(12 h)	F(24 h)	E + F(24 h)
*DEFB103A*	ILMN_1684308	β-defensin-3	Antimicrobial	1.40	1.41	2.17	2.02	7.00
*DEFB4A*	ILMN_2048043	Β-defensin-2	Antimicrobial	0.98	2.35	5.11	2.94	8.43
*LCN2*	ILMN_1692223	Lipocalin-2	Antimicrobial	2.18	6.38	9.47	4.89	9.87
*RNAse7*	ILMN_1712849	Ribonuclease, RNAse A family, 7	Antimicrobial	1.46	1.33	1.97	2.08	5.41
*SLPI*	ILMN_2114720	Secretory leukocyte peptidase inhibitor	Antimicrobial	2.16	1.54	3.16	—	2.79
*WFDC5*	ILMN_2079042	WAP-four-diS core domain-5	Antimicrobial, Protease Inhibitor	—	—	3.17	—	2.94
*S100A7*	ILMN_1757351	S100 calcium binding protein A7/Psoriasin	Antimicrobial, Keratinisation, Inflammation	1.86	1.48	5.07	2.50	10.66
*S100A8*	ILMN_1729801	S100 calcium binding protein A8	Keratinisation, Inflammation	2.03	2.24	3.39	2.10	2.41
*S100A9*	ILMN_1750974	S100 calcium binding protein A9	Keratinisation, Inflammation	2.13	2.53	5.86	2.25	3.79
*S100A12*	ILMN_1748915	S100 calcium binding protein A12	Antimicrobial, Keratinisation, Inflammation	1.13	1.22	2.69	—	5.23
*CNFN*	ILMN_1803838	Cornifelin	Keratinisation	2.04	1.30	5.90	—	6.50
*LCE3D*	ILMN_1718395	Late cornified envelope 3D	Keratinisation	2.11	1.64	5.76	2.64	10.37
*SBSN*	ILMN_1712759	Suprabasin	Keratinisation	2.06	1.35	4.44	—	5.25
*SPRR2A*	ILMN_1795359	Small proline-rich protein 2A	Keratinisation	2.06	3.95	14.75	3.60	17.04
*SPRR2C*	ILMN_2197577	Small proline-rich protein 2C	Keratinisation	1.11	1.60	5.77	2.77	19.52
*SPRR2D*	ILMN_2191967	Small proline-rich protein 2D	Keratinisation	1.98	2.09	5.73	—	4.10
*SPRR2E*	ILMN_2211018	Small proline-rich protein 2E	Keratinisation	1.73	3.14/2.46	13.53/10.84	2.43	11.75
*SPRR2F*	ILMN_1674367	Small proline-rich protein 2F	Keratinisation	2.20	3.60	13.08	2.79	12.32
*SPRR2G*	ILMN_1702127	Small proline-rich protein 2G	Keratinisation	1.26	1.81	7.27	3.25	22.46
*KRT1*	ILMN_1735712	Keratin 1	Keratinisation	—	—	8.82	—	5.13
*KRT6C*	ILMN_1754576	Keratin 6C	Keratinisation	—	—	2.95	—	2.50
*IL36G*	ILMN_2158713	Interleukin 36 gamma	Inflammation	1.00	2.70	4.34	2.08	4.05
*SERPINB4*	ILMN_1782716	Serpin peptidase inhibitor clade B, member 4	Inflammation	1.03	2.92	6.48	—	3.42
*CXCL10*	ILMN_1791750/1791759	Chemokine ligand 10	Inflammation	—	41.73	16.00	9.82	6.46
*CCL5*	ILMN_2098126/1773352	Chemokine (C-C motif) ligand 5	Inflammation	—	9.60/6.30	4.56/3.12	4.22	3.04

### VK2 E6/E7 cell culture and challenges

VK2 E6/E7 vaginal epithelial cells were cultured in Keratinocyte SFM media (Life Technologies) at 37 °C and 5% CO_2_. Cells were grown in 75cm^2^ flasks (Corning) before being passaged and seeded into either 6-well (5 × 10^5^ cells), 12-well (1.2 × 10^5^ cells) or 96-well plates (1 × 10^5^ cells). To model patient vaginal estrogen treatments cells were pre-treated with either 4 nM estrogen ((2-hydroxypropyl)-β-cyclodextrin encapsulated17β-estradiol) or cyclodextrin (vehicle control) equivalent (Sigma) for 7 days before challenge with either 50 ng/ml flagellin isolated from *E. coli*^17^ for up to 24 hours or phosphate buffered saline (PBS). Cell culture medium, vehicle control and estrogen were typically replaced every 48 hours.

VK2 cells were seeded into 12-well plates at a density of 1 × 10^5^ cells/well in keratinocyte-SFM media and pre-treated with either 4 nM 17β-estradiol or 14.8 nM cyclodextrin for 7 days prior to challenge. At 80% confluency the cells were washed with PBS, 1 ml keratinocyte-SFM medium added and after 24 hours the cells challenged with 100 ng/ml IL-17A (Sigma, USA), or 100 μM Tris-HCL, pH 7.4 with 2.5 mM NaCl, and 50 ng/ml flagellin (minimum concentration of flagellin shown to induce an innate response) or PBS. After 24 hours the medium was removed, the cells washed with PBS, the RNA extracted using the SV Total RNA Isolation Kit (Promega) as per manufacturer’s instructions and RNA samples stored at −80 °C.

### Microarray and qPCR analysis

VK2 E6/E7cells were treated with as follows: cyclodextrin (vehicle control) for 7 days plus/minus 12 h flagellin (50 ng/ml) challenge, estrogen (4 nM) for 7 days plus/minus 12 h flagellin challenge. RNA was extracted from the cells using the SV Total RNA Isolation Kit (Promega) as per manufacturer’s instructions and quality verified using an Agilent 2100 Bioanalyser. Samples were sent to Service XS (Netherlands) for microarray analysis using the Illumina HumanHT-12 v4 Expression Beadchip. Raw data were converted into fold change relative to control and presented as heatmaps in collaboration with the Newcastle University Bioinformatics Support Unit using the R software platform with Bioconductor^20,21^. Microarray data has been deposited in the Gene Expression Omnibus Database, accession number GSE135583. Genes were considered significantly different if a fold change ≥ +2 or −2 and P < 0.05 was recorded.

Biopsy RNA was also extracted using the SV Total RNA Isolation Kit (Promega) and RNA samples stored at −80 °C. Nucleic acid was quality checked and quantified by nanodrop as previously described^17^. Reverse transcription of RNA (400 ng) was performed using random hexamers (Roche), MMLV Reverse Transcriptase and RNase Inhibitor (Promega). Gene expression was quantified by qPCR using SYBR Green (Roche) with 0.5 μm primers on a LightCycler 480 (Roche) with each plate containing controls for verification of single target amplification using melt curve technology^22^. Data were normalised to the appropriate reference genes *GAPDH* and *ATP5b*^17^; primer sequences of target genes are shown in Table 2S.

### Epithelial estrogen receptor analyses

VK2 E6/E7 cells seeded at a density of 1 × 10^5^ cells/well were pre-treated with either 1 µM ICI 182,780 known as fulvestrant (Tocris Bioscience) or 0.1% DMSO vehicle and 4 nM β-cyclodextrin encapsulated 17β-estradiol or cyclodextrin vehicle for up to 7 days. The culture medium, ICI 182,780, encapsulated 17β-estradiol or cyclodextrin were replenished every 48 hours and 24 hours before a flagellin 50 ng/ml or PBS challenge.

For G-protein coupled estrogen receptor (GPER) stimulation VK2 E6/E7 cells seeded at a density of 1 × 10^5^ cells/well were pre-treated with either 100 nM GI or 0.01% DMSO vehicle and 4 nM β-cyclodextrin encapsulated17β-estradiol or cyclodextrin vehicle 24 hours before a flagellin 50 ng/ml or PBS challenge. The culture medium, 17β-estradiol or cyclodextrin were replenished every 48 hours.

### ELISA host defence peptide analyses

Vaginal fluid samples were analysed for hBD2, hBD3, LCN2 and SLPI by ELISA. hBD2 ELISA (Peprotech) – capture antibody 100 µg/ml, detection antibody 0.5 µg/ml, samples diluted 1:1; hBD3 ELISA – capture antibody 0.25 µg/ml (Abcam), detection antibody 1 µg/ml (Abcam), samples diluted 1:4; LCN2 ELISA (R&D systems) – capture antibody 2 µg/ml, detection antibody 25 ng/ml, samples diluted 1:49; SLPI ELISA (R&D systems) – pre-coated plates, as per manufacturer’s instructions, samples diluted 1:79. Each plate included a standard curve and negative control. Plates were read using a FLUOstar Omega plate reader (BMG Labtech, Germany) at 450 nm with correction at 540 nm and blank-corrected values analysed using 4-parameter curve fit.

### ERE site directed mutagenesis and luciferase reporter assay

A 2030 bp sequence upstream of the *DEFβ4* gene ATG start codon cloned into plasmid phBD-2-Luc (Promega) was used in analysing roles of putative estrogen response elements (ERE). Point mutations of putative EREs were generated using customised primers, a QuikChange II site-directed mutagenesis kit (Agilent, USA) (Table 2 S) and confirmed by sequencing (GeneVision, UK). Transfection of plasmids into VK2 E6/E7 cells was performed using the reverse transfection method. Briefly, 150 ng DNA and 0.5 µl Attractene (Qiagen) were added to 50 µl media (per well) and left for complex formation for 15 minutes. Cells were plated into 96-well clear bottomed plates at a density of 5 × 10^4^ cells/well in media supplemented with 4 nM 17β-estradiol or 14.8 nM cyclodextrin. The plasmid/attractene complex was added and the cells left to adhere over-night. Following a media change, the cells were incubated for a further 24 hours and challenged with either flagellin (50 ng/ml) or PBS. Post-challenge, cells were lysed as per the Luciferase Assay System protocol (Promega) and luminescence read using a FLUOstar Omega Microplate reader (BMG Labtech).

### Statistical analyses

Statistical analyses were performed using GraphPad Prism 6 software. Following data analyses by one or two way ANOVA multiple comparisons were performed using Dunnet’s, Sidak’s or Tukey’s post hoc tests.

## Results

### Estrogen and flagellin treatment of VK E6/E7 cells - microarray data

**Figure 1:**
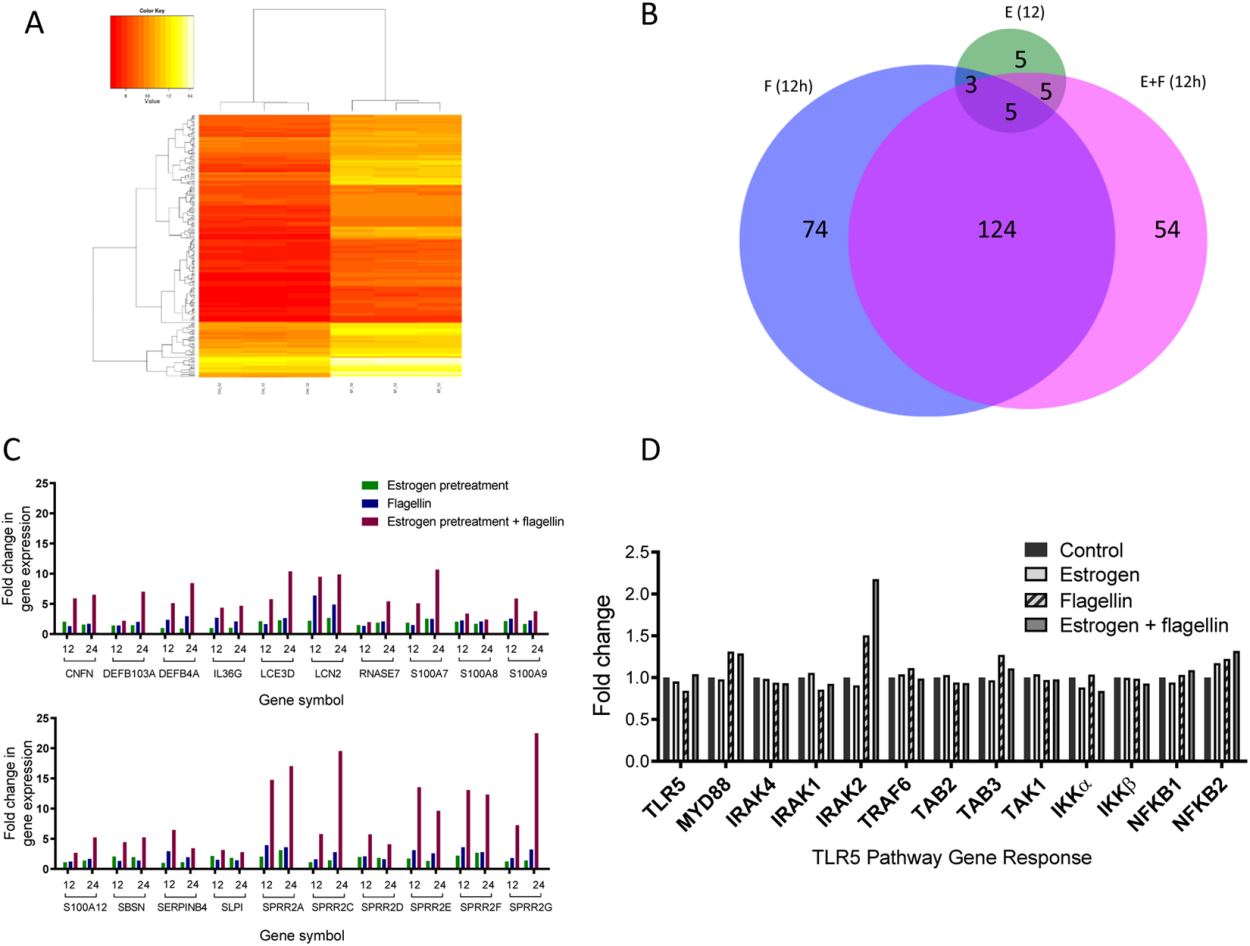
(**A**) Heatmap showing differential gene expression patterns following 12 h treatments of VK2 E6/E7 cells with vehicle (ctrl_12) (n = 3) and estrogen + flagellin (EF_12) (n = 3). Rows represent individual genes and their fold expression measurements while columns show different experimental conditions; genes are clustered according to patterns of expression and functionality (**B**) Venn Diagram illustrating differentially regulated genes following pre-treatment of VK2 E6/E7 cells with E (Green), F (12 h) (Blue) and E + F (12 h) (Pink). Genes upregulated in response to E + F but not E or F were *KRT1, SPRR2G, SPRR2C, S100A7, DSG1, SERPINB3, S100P, TMEM45A, DSC1 (2 probes), TMEM45A, NCCRP1, WFDC5, KRT6C, KRTDAP, CYP4F22, ALOX12B, ZNF750, ID1, SPRR2B, SPRR1A, S100A12, FABP4, ALDH3B2, LOR, KRT80, IGFBP3, SLC5A1, KLK11, CXCL14, CLDN1, SPRR1B, IGFBP3, KRT10, EGR1, TSC22D3, TSC22D3, SIAE, THBS1, MAFB, KLK12, DEFB103A, SULT2B1, A2ML1, ZC3H12A, CRCT1, SPRR3, DLX5, TGM1, GJB2, HOPX, F3, KLK7, SULT2B1;* Five genes each upregulated in response to E and F and E + F treatments were *LCN2, S100A8, S100A9, SPRR2A, SPRR2F;* (**C**) Microarray gene expression data (fold changes) relating to E pretreatment (green), F 12 & 24 h challenges (blue) and E + F12 & 24 h F challenges (red); (**D**) Expression of genes encoding TLR5 signalling pathway molecules: vehicle treated/control (black), estrogen (**E**) (light grey), flagellin (**F**) (diagonal stripes) and estrogen + flagellin (E + F) (dark grey).

Microarray data relating to the E, F (12 h) and E + F (12 h) challenges were mined and gene probes shown to be differentially expressed in response to either estrogen pretreatment (E), or Flagellin (F12) and/or E + F12 treatments identified (Fig. 1A & B). Ontology analyses of these 270 probes identified up-regulation of expression pathways specifically related to innate immunity, cell differentiation and keratinization.

Notably five genes (Fig. 1B), *LCN2*, *S100A8, S100A9, SPRR2A* & *SPRR2F* encoding proteins involved in host defence and keratinisation were differentially expressed in response to each of the three treatments (E12, F12, and E + F12). All genes were upregulated (Fig. 1C) with the highest level of expression observed in response to E + F12. These data indicated that estrogen pretreatment of the VK2 E6/E7 cells prior to infection (F challenge) enhanced gene expression and augmented the host response.

F (12 h) and E + F (12 h) treatments significantly affected the expression of 124 common gene probes (Fig. 1B). Within this group were six gene probes that showed significantly fold higher expression following E + F12 treatment compared to F12h treatment alone. Such genes included *SPRR2D* (5.73 v 2.09), *SPRR2E* (two probes) (10.8 v 2.4; 13.53 v 3.14), *DEFβ4* (2.35 v 5.11), *IL36G* (4.34 v 2.7) and *SERPINB4* (6.48 v 2.92) encoding peptides and proteins involved in vaginal keratinisation and host defence including the inflammatory response (Table 1). However, downregulation of gene expression was also detected in response to the E + F12 treatment. Examples included genes encoding the chemokines CXCL10 and CCL5 involved in the inflammatory response, which showed fold changes of 41.73 and 9.6, respectively, after F12 treatment but 16 and 4.56, following E + F12 treatment (Table 1).

Five gene probes were shown to be upregulated by E and E + F12 but not F12 treatments. Genes represented were *CNFN, F3, LCE3D, SBSN and SLPI*, which apart from F3, again encoded proteins linked to cell keratinisation, differentiation and host defence. These data supported estrogen functioning naturally to augment the cell defences as well as during periods of infection.

Data mining also identified 54 gene probes that were differentially expressed in response to E/F12, but neither E nor F12 treatments (Fig. 1B). All 54 probes showed significant upregulation and reflected molecules involved in host defence including, *S100A7* (5.07 fold); *WFDC5* (3.17); inflammation, *CXCL14* (2.45) as well as cell differentiation and keratinisation *SPRR2C* (5.77), *SPRR2G* (7.27), *KRT1* (8.82 fold), *KRT6C* (2.95).

When the F challenges were repeated for 24 h rather than 12 h comparable changes in differential gene expression were observed (Figure S1A & B) although variations were detected. For example increased expression of *RNASE7* (1.9 v 5.4 fold), *SPRR2C* (5.77 v 19.5), and *SPRR2G* (7.27 v 22.6) were detected at 24 h following the E + F treatments (Fig. 1C; Table 1).

UPEC are characterised by flagella and the host Toll-like Receptor 5 (TLR5) pathway is key in detecting potential uropathogens and activating the urogenital innate defences^17,23^. Microarray data focussed on the TLR5 signalling pathway suggested E, F12, E + F12 treatments did not affect expression of genes encoding proteins involved in TLR5 signalling except for the *IRAK2* gene which was upregulated (>2 fold) in response to the E + F treatment (Fig. 1D).

### Topical vaginal estrogen treatment of post-menopausal women - *in vivo* data

**Figure 2:**
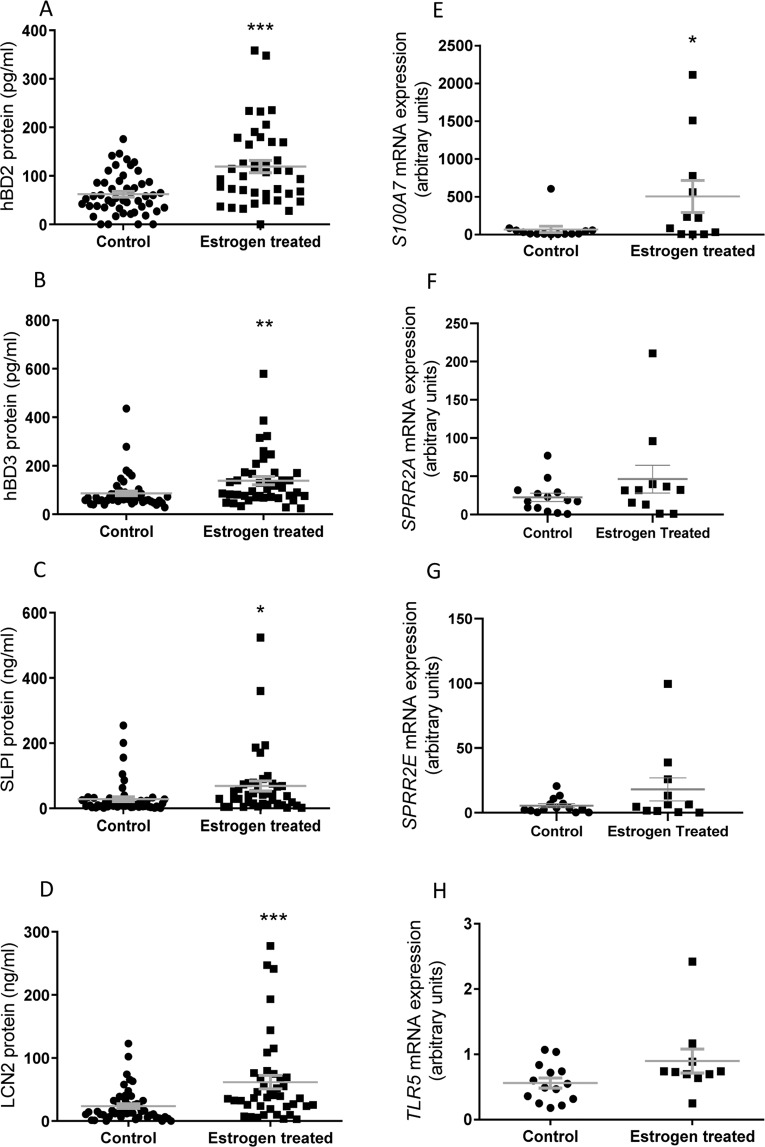
Peptide concentrations of host defence agents measured in vaginal douche samples from control and estrogen treated patients (**A**) hBD2; (**B**) hBD3; (**C**) SLPI (**D**) LCN2 [n = 91, C = 50;E = 41]. Gene expression relating to (**E**) *S100A7* (**F**) *SPRR2A* (**G**) *SPRR2E* (**H**) *TLR5*. mRNA expression presented as relative expression in vaginal biopsies from control and estrogen treated patients [n = 25, C = 14; E = 11]. * P < 0.05; **P < 0.01; *** P < 0.001.

To verify the physiological significance of these *in vitro* data vaginal douche or biopsy samples from control and post-menopausal women using topical vaginal estrogen were analysed. Results (Fig. 2A–D) indicated that topical estrogen treatment increased the vaginal concentrations of host defence agents including LCN2 and SLPI, which supported the microarray data, but significant increases in the concentrations of the defensins hBD2 and hBD3 (P < 0.05), were also observed. These latter observations were arguably more analogous to the E + F *in vitro* treatments potentially reflecting the vaginal responses to the natural urogenital microbiota. Analyses of vaginal biopsies also supported increased *S100A7* gene expression indicative of enhanced barrier integrity (P < 0.05) (Fig. 2E) but surprisingly *SPRR2A and SPRR2E* expression, indicative of keratinocyte cell differentiation, were comparable between the two cohorts (Fig. 2F,G). Analysis of patient vaginal biopsies for *TLR5* gene expression revealed no significant differences between the control and estrogen treated cohorts (Fig. 2H).

### VK2 E6/E7 estrogen receptors and innate effector gene regulation

**Figure 3:**
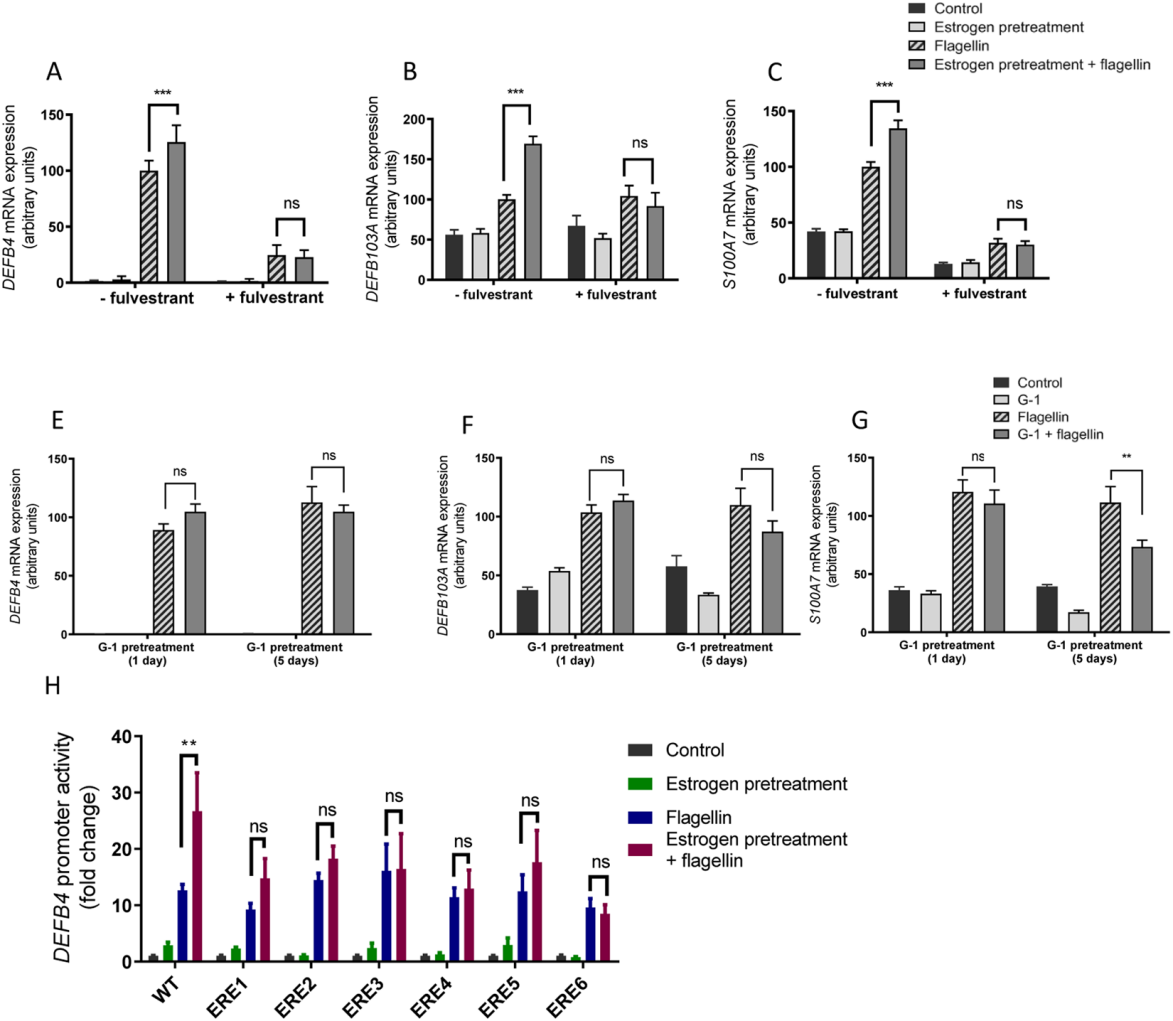
Effects of fulvestrant treatment on *DEFβ4* (**A**) *DEFB103A* (**B**) and *S100A7* (**C**) expression in VK2 E6/E7cells pretreated with estrogen, challenged with F or E + F [N = 3; n = 9]. Effects of G1 treatment on *DEFβ4* (**D**) *DEFB103A* (**E**) and *S100A7* (**F**) expression in VK2 E6/E7 cells pretreated with estrogen, challenged with F or E + F [N = 2; n = 6]. (**H**) Effects of mutating EREs in the *DEFβ4* promoter measured by luciferase activity and data normalised to a cyclodextrin-treated control [N = 4; n = 8]. Data presented as mean ± SEM. ns – not significant, **P < 0.01; *** P < 0.001.

Estrogen functions through either a classical pathway whereby estrogen receptors interact with estrogen responsive elements (ERE) in the target gene promoter or through a non-classical mechanism involving the membrane estrogen receptor GPER^24^. VK2 E6/E7 cells express genes encoding the classical intracellular ESR1 (ER-α) and ESR2 (ER-β) steroid receptors and the G protein-coupled estrogen receptor (GPER) (confirmed by end-point PCR and sequencing). Incubating estrogen treated VK2 E6/E7 cells with fulvestrant (1 µM) to inhibit ESR1/2 functioning and challenging with F (50 ng/ml) inhibited the augmenting effects of estrogen on effector gene expression, although interestingly gene expression in response to UPEC flagellin was also markedly reduced (Fig. 3A–C).

Fulvestrant functions as an antagonist of ESR1/2 activity, but is also an agonist of GPER. Incubating estrogen treated cells with 100nM G-1, a selective GPER receptor agonist with no activity against ESR1/2 receptors and challenging with F did not impact effector gene expression (Fig. 3E–G).

The host defence peptide HBD2 encoded by the *DEFβ4* gene plays a significant role in the urothelial innate defences^17^. Putative ERE response elements were identified in the 5’ regulatory region of the gene (Fig. 2S) so this gene was targeted for further investigation of the transcriptional mechanism. Challenging VK2 E6/E7 cells, transfected with 2Kbp of 5’ *DEFβ4* gene regulatory sequence fused to a luciferase reporter, with flagellin resulted in a 12-fold increase in luciferase activity that increased to 27-fold following estrogen pre-treatment of the cells. Mutation of these putative ERE sites (Fig. 2S) inhibited any potentiation of reporter activity (Fig. 3H), but statistical analyses of the WT (E + F) versus ERE1-6 mutation data did not support any redundancy, ERE 4 (P = 0.07) and ERE 6 (P = 0.055).

### Estrogen and transcriptional regulation of the innate IL-17 defences

**Figure 4:**
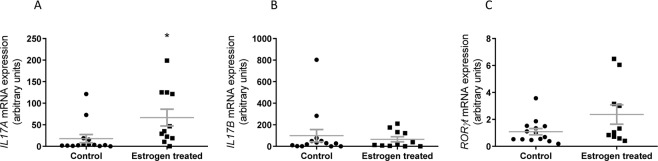
*IL-17A* (**A)** *IL-17B* (**B)** *RORγt* (**C)**, mRNA expression presented as relative expression in vaginal biopsies from control and estrogen treated patients [n = 25, C = 14; E = 11]. Data presented as mean ± SEM. *P < 0.05.

Ingenuity pathway analyses of the microarray data highlighted ‘The role of IL-17A in Psoriasis’ as the canonical pathway most activated following treatment of VK2E6/E7 cells with E + F (Table S3). This pathway describes IL-17A binding to the IL-17 receptor complex (ILRA/RC) and transcription of genes including *DEFβ4* and *S100A7*. Interestingly the vaginal biopsies of the estrogen treated post-menopausal subjects were characterised by increased (P < 0.05) IL-17A, but not IL-17B expression (Fig. 4A,B). Residing within skin and mucosal epithelia are populations of innate T cells that produce IL-17 during periods of infection^25^. Expression (p = 0.06) of the transcription factor *RORγt* gene (Fig. 4C), in the estrogen treated vaginal biopsies did not, however, support increased numbers of vaginal IL-17 producing T cells.

**Figure 5:**
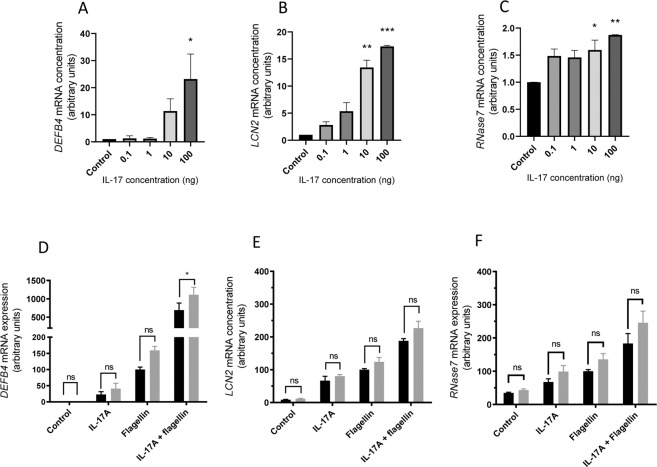
Effects of IL-17A peptide challenges on DEFβ4 (**A**), LCN2 (**B**) and RNase7 (**C**) gene expression in VK2 E6/E7 cells. Effects of IL-17A peptide 100 ng/ml on DEFβ4 (**D**) LCN2 (**E**) and RNAse7 (**F**) expression in VK2 E6/E7 pretreated with cyclodextrin ■ or estrogen  and challenged with F/IL-17A [N = 2; n = 6]. Data presented as mean ± SEM. ns – not significant; *P < 0.05; **P < 0.01; ***P < 0.001.

VK2 E6/E7 vaginal cells express the genes encoding IL-17 receptors, confirmed by PCR and DNA sequencing, and responded directly to recombinant IL-17A (0.1 to 100 ng/ml) through increased (P < 0.05) expression of genes encoding host antimicrobials (Fig. 5A–C). Estrogen pre-treatment of VK2 E6/E7 cells did not enhance these IL-17A effects, but synergistic effects were suggested when mimicking infection through the use of flagellin (Fig. 5D–F).

## Discussion

The female climacteric is characterised by decreased circulating concentrations of estrogen, which in some women, coincide with an increased susceptibility to rUTIs peri and post menopausally. Topical vaginal estrogen helps reduce UTI incidence^1,18^ with the protective mechanisms being multifaceted and involving alterations in vaginal pH and microbiota composition^15,26^, the strengthening of the bladder epithelial barrier and the enhanced antimicrobial capacity of the urothelium^16,17^. Our clinical and *in vitro* data also supported topical estrogen as functioning to protect the vaginal epithelium through increased production of innate effector molecules and barrier strengthening, but furthermore suggested that estrogen helps boost these vaginal defences through augmentation of the TLR5 response to flagellin. Arguably this enhanced innate response reduces microbial colonisation of the vaginal regions and helps protect against flagellated microbes such as UPEC causing UTIs.

UTIs are associated with bacterial motility and flagellated uropathogens such as UPEC ascending into the bladder and causing UTIs^27^. Host urothelial cells are characterised by TLR5 receptors that detect UPEC flagella and their activation orchestrates the synthesis, and release of host innate effectors including cytokines and antimicrobials, which kill potential uropathogens^17,28^. Vaginal cells, characterised by both ESR and GPER proteins also exhibit TLR5 receptors^29,30^ suggesting that the estrogen associated boost in the vaginal TLR5/F effector responses results from either direct or indirect physical interactions between the different receptor types. Inhibitor studies supported the involvement of ESR1/2, but not the membrane located GPER, and suggested a direct molecular mechanism of gene regulation. This was confirmed by mutagenesis of putative ERE sites in the *DEFβ4* promoter region. These data also supported ESR1/2 binding and NFκB signalling, the cellular response to flagellin challenge^17^, functioning synergistically to boost the vaginal innate defences. A comparable synergistic response, again involving *DEFβ4* expression, but linked to lipopolysaccharide challenge has also been reported in primary vaginal cells pre-treated with estrogen^31^.

Promoters of other genes known to encode agents involved in the urogenital innate defences and including *LCN2, RNASE7* and *S100A7* are also characterised by putative EREs (Fig. 2S). Assuming similar regulatory mechanisms operate this illustrates, potentially, how reduced circulating estrogen concentrations associated with the climacteric associate with diminished epithelial defences, which expose the vaginal tissues to bacterial colonisation and/or repeated infections. ESR knockout mouse studies, which reported the reduced production of secretory proteins, including the host defence agent lactoferrin in the uterine epithelial tissues^32^, suggest ESR1 (ERα) activity to be key in the response. However, there is uncertainty as others have reported ESR2 (ERβ) as being more important in regulating the bladder antimicrobial response^16^.

It has been reported that estrogen treatment of vaginal epithelial cells (48 h) associates with decreased secretion of the host defence molecules, hBD2 and elafin^33^. These observations are difficult to explain physiologically as decreased vaginal hBD2 concentrations are characteristic of post-menopausal women^17^. Moreover impaired elafin expression is related to stress urinary incontinence and pelvic organ prolapse, conditions again more reflective of women with low circulating estrogen concentrations^34^. One explanation for these anomalies relates to the cell models used, that is, freshly isolated vaginal cell preparations compared to vaginal biopsies or robust cell monolayers.

TLR5 is a key host factor in detecting potential UPEC infections^17,28^. Reports using human bladder T24 cells suggest that estrogen treatment is linked to TLR5 suppression with prolonged flagellin exposure further reducing TLR5 protein concentrations as measured by ELISA^35^. In contrast, our VK2 E6/E7 microarray data analysed in response to estrogen and E + F treatments did not suggest changes in TLR5 expression. Although paradoxical these two sets of data are consistent with receptor activation and dimerization, and the potential loss of antigenic binding sites available to TLR5 antibodies, which could be interpreted as reduced receptor protein. The vaginal biopsy data also revealed no changes in TLR5 gene expression in women using topical estrogen although expression has been previously shown to be significantly increased in post-menopausal women experiencing active UTIs^17^. These latter observations suggest factors other than estrogen and flagellin are involved in regulating TLR5 expression *in vivo*.

Similar to published data^18,19^ the microarray analyses suggested that expression of a number of genes encoding proteins involved in vaginal barrier integrity and thickening eg *SPRR2A, SPRR2F*, *LCE3D* and *CNFN* were significantly enhanced in response to estrogen treatment. Importantly our data also showed that expression of gene subsets, including those encoding proteins involved in keratinisation such as the small proline rich proteins (Table 1), were further enhanced in response to flagellin, used experimentally to mimic an UPEC infection. These observations supported synergistic mechanisms functioning to help protect the integrity and barrier structure of the vaginal epithelium. It is of interest that peptide fragments called keratin-derived antimicrobial peptides (KDAMPs), have also been reported to act as antimicrobial agents in epithelial cells^36^. Vaginal estrogen treatments may therefore not only enhance resistance to infection through direct modifications of the epithelial barrier, but also through increased KDAMP production, hence augmenting the more traditional epithelial host innate defences.

Ingenuity pathway analyses of the microarray data highlighted a potential role for IL-17 in protecting the vaginal epithelium from microbial infections. This pro-inflammatory cytokine is linked to populations of innate-like lymphocytes including γδ T cells located within epithelial barriers^37^ and functions through direct induction of epithelial host defence agents and increased neutrophil recruitment^25,38^. While studies in mice have shown that vaginal fungal challenges are associated with increases in vaginal IL-17 concentrations that signal through epithelial IL-17R to induce host defence peptides^39^ less is known of the roles, if any, of vaginal IL-17 in protecting against bacterial mediated UTIs. However, the fact that vaginal biopsies from post-menopausal women treated with topical estrogen hinted of increased T cell numbers, potentially γδ T cells, and IL-17A but not IL-17B gene expression, indicates a significant vaginal innate defence mechanism whose role(s) need further exploration in relation to UTI pathologies. Moreover the observation that the VK2 E6/E7 antimicrobial responses modelled *in vitro* using combinations of IL-17 and UPEC flagellin, could be enhanced in the presence of estrogen, again demonstrated the promiscuity of the steroid hormone in boosting the vaginal innate defences.

For flagellated uropathogens such as UPEC transiting from the gut to the bladder, the vaginal epithelium provides a potential colonisation site. These data show how estrogen plays key roles in directly enhancing a plethora of vaginal innate defence mechanisms that encompass epithelial integrity and antimicrobial potency and function collectively, to reduce or prevent microbial colonisation. These observations also help explain why post-menopausal women characterised by reduced estrogen concentrations are at increased risk of rUTIs associated with flagellated UPEC.

## Supplementary information

Supplementary Information.
