# Supplementary material for: Topical Estrogen Treatment Augments the Vaginal Response to Escherichia coli Flagellin
Source: Sci Rep. 2020 May 21;10:8473. doi: 10.1038/s41598-020-64291-y (PMC7242342; doi:10.1038/s41598-020-64291-y)
Supplement: Supplementary file 1 — Supplementary Information. [file 41598_2020_64291_MOESM1_ESM.pdf]

Topical Estrogen Treatment Augments the Vaginal Response to *Escherichia coli* Flagellin

Authors:

Anna Stanton, Catherine Mowbray, Marcelo Lanz, Karen Brown, Paul Hilton, Alison Tyson-Capper, Robert S Pickard, Ased SM Ali, Judith Hall

Figure S1: Venn Diagram illustrating differentially regulated genes following pre-treatment of VK2 E6/E7 cells with Estrogen (E) (Green), Flagellin (F) (24h) (Blue) and E+F (Pink).

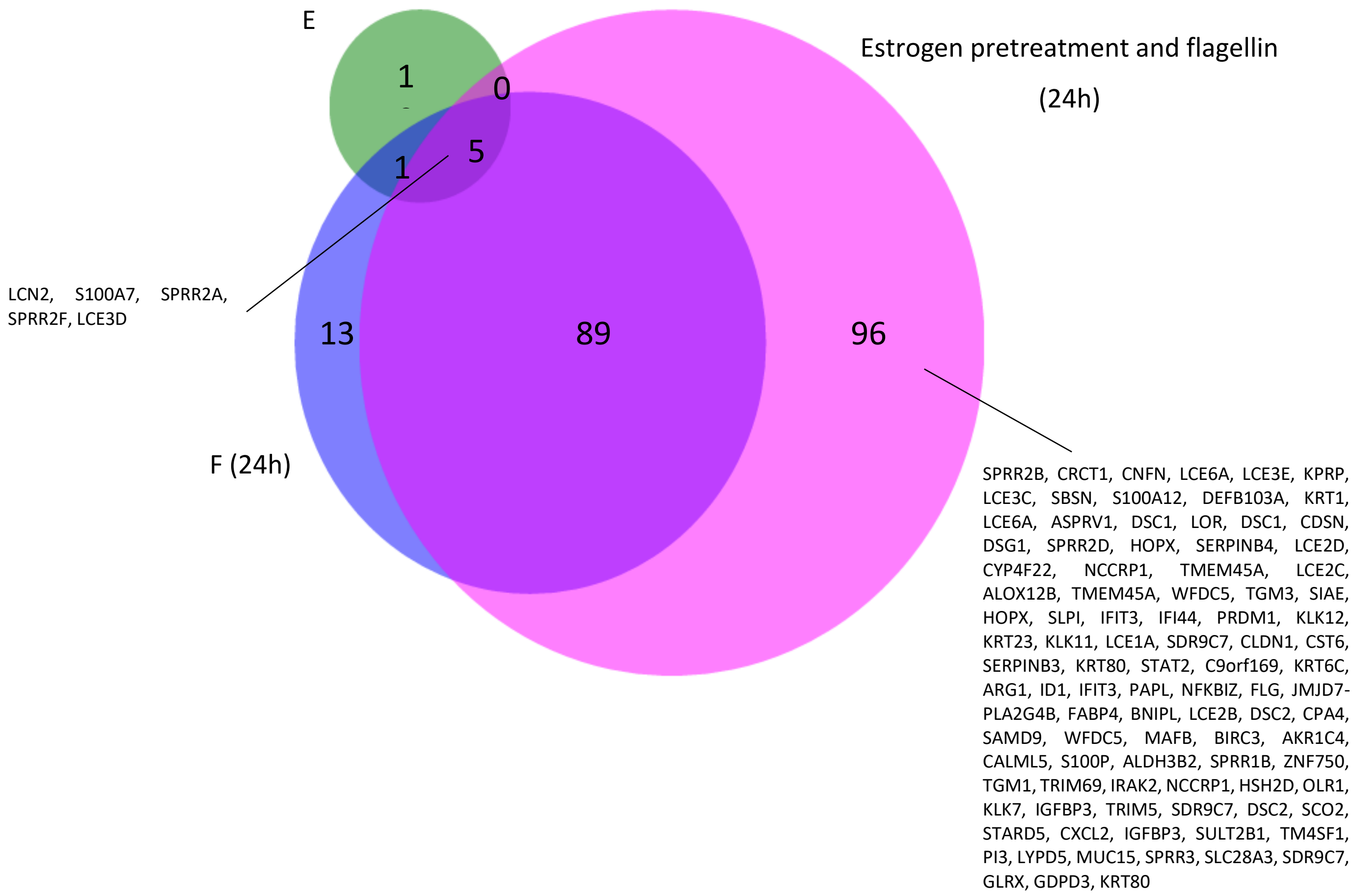

Figure S1: Heatmap showing differential gene expression patterns following 24 h treatments of VK2 E6/E7 cells with vehicle (ctrl_24) (n=3) and estrogen + flagellin (EF_24) (n=3). Rows represent individual genes and their fold expression measurements while columns show different experimental conditions; genes are clustered according to patterns of expression and functionality

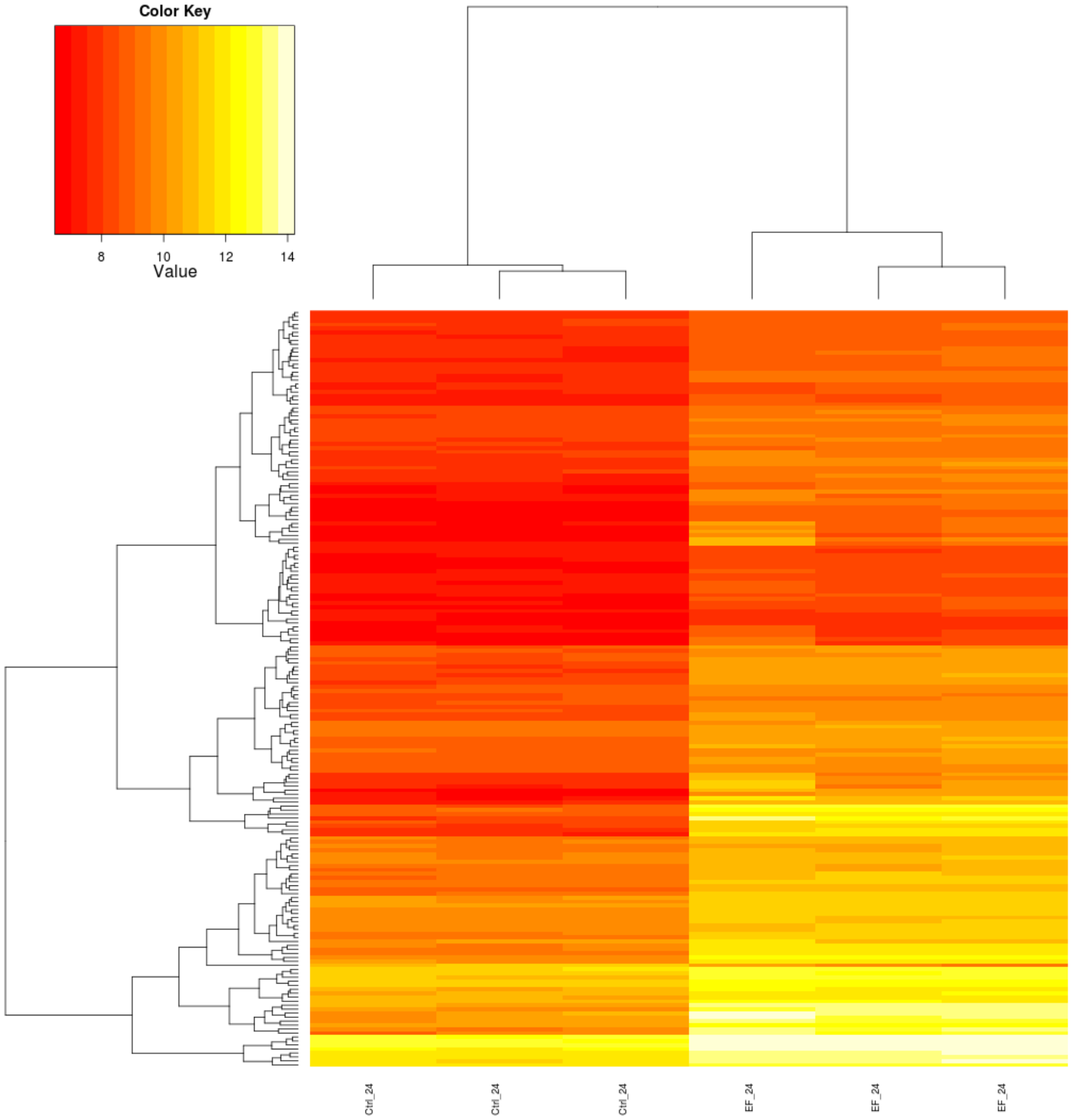

A: DEFβ4

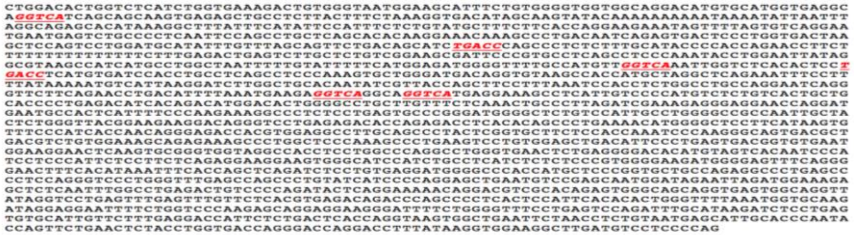

DEFβ4 5' ERE Mutations:

ERE1: ATGGCGAGGCAAATTACAGCAGCAAG

ERE2: TGACAGCATGTATCTCAGCCTCTCTTTG

ERE3: TTGCCATGTTAGTGAAATTGGTCTCACA

ERE4: CTCACACTCCTATCTTCATGTGATCCACC

ERE5: AATGAAGAAATTAGGCAGGTC

ERE6: GGTCAGGCAAATTATGAGGAAAGCCT

B: LCN2

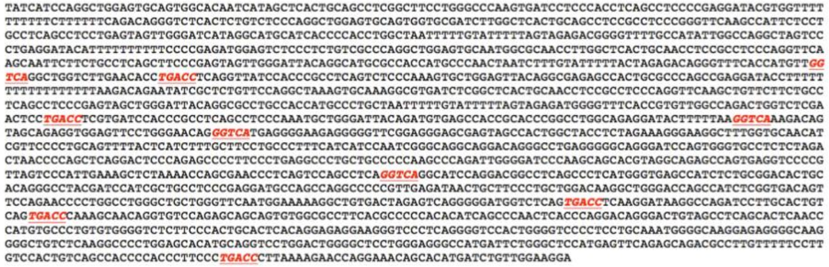

C: RNase 7

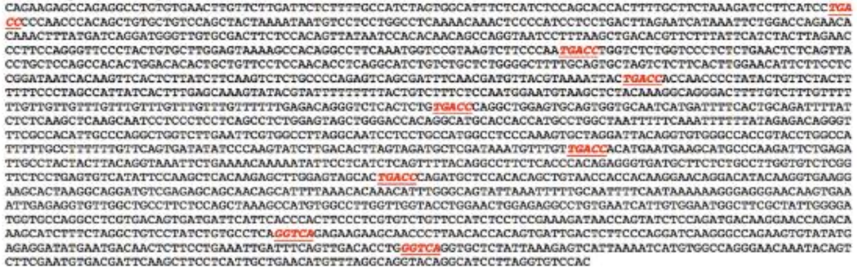

D: S1000A7

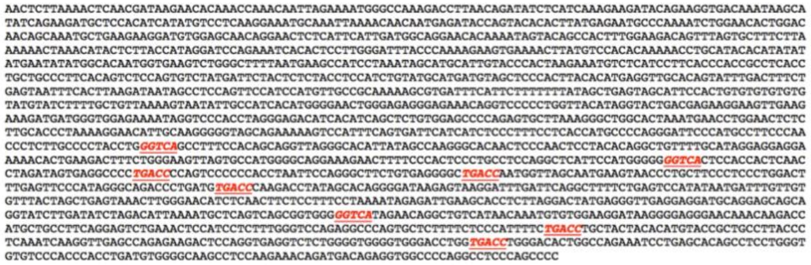

Figure 2S: Potential EREs (highlighted in red) in the 2Kb promoter regions of A: *DEFβ4*, B: *LCN2*, C: *RNase7* and D: *S100A7* genes as identified by PROMO software. Mutations of each of the six putative ERE half-sites in the 2032bp *DEFβ4* promoter region (ERE1-6) are underlined.

	Control	Estrogen Treated
Number	53	41
Median Age<br>(Range)	60y<br>(47-78y)	57y<br>(46-81y)
Taking vitamins	24	18
Previous HRT/estrogen treatments*	34	10
Previous STI	0	0
Previous urological or gynaecological surgery	34	18
Median no of pregnancies<br>(Range)	2<br>(0-6)	2<br>(0-4)

Table 1S: Patient Clinical History.

* None of the patients had been prescribed or used estrogen in the previous six months to the study.

Table 2S: qPCR and mutagenesis primers

Primer	Sequence	Annealing Temperature (°C)	Product Size (bp)
S100A7 F	ACACCAGACGTGATGACAAG	58	114
S100A7 R	CATCGGCGAGGTAATTTGTG		
SPRR2A F	ACACAGGGAGCTTCTTTCTCC	56	126
SPRR2A R	CCAGGACTTCCTTTGCTCAGT		
SPRR2E F	AAGGAAGTCCTCAGCAGTGT	56	142
SPRR2E R	AATTGCACAGGTGTTAGAAGCC		
TLR5 F	TGATGTTCATGTTCCTGACACT	57	91
TLR5 R	AGCATCCCTGGTTTGGTGAC		
DEFB4 F	CAGCCATCAGCCATGAGGGT	58	83
DEFB4 R	CCACCAAAAACACCTGGAAGAGG		
DEFB103AF	GTGAAGCCTAGCAGCTATGAG	60	89
DEFB103AR	TGATTCCTCCATGACCTGGAA		
IL17A F	TGGAATCTCCACCGCAATGA	59	130
IL17A R	GCTGGATGGGGACAGAGTTC		
IL17B F	CTCAGCTACGACCCAGTGC	60	100
IL17B R	CGTGTCCACACGGTATCTCC		
RORγt F	GCTTCTCAAAGCAGGAGCAA	58	108
RORγt R	AGCTCCATGCCACCGTATTT		
LCN2 F	CAAAGACCCGCAAAAGATGT	58	128
LCN2 R	GGCAACCTGGAAGAAAAGTC		
RNase7 F	TGACAGCCTAGGAGTGCGT	56	83
RNase7R	CAGGGGTCGCTTTGCG		
DEFβ4			
ERE1 F	GTGCATGGTGAGGCAAATTATCAGCAGCAAGTGAGAGCT	55	
ERE1R	AGCTCTCACTTGCTGCTGATAATTTGCCTCACCATGCAC		
ERE2F	GTTCTGACAGCATCTATCTCAGCCCTCTCTTTGCATACC	55	
ERE2R	GGTATGCAAAGAGAGGGCTGAGATAGATGCTGTCAGAAC		
ERE3F	GGTTTTGCCATGTTAGTGAATTGGTCTCACA	55	
ERE3R	TGTGAGACCAATTCACTAACATGGCAAAACC		
ERE4F	CAAATTGGTCTCACACTCCTATCTTCATGTGATCCACCTGCCT	55	
ERE4R	AGGCAGGATCACATGAAGATAGGAGTGTGAGACCAATTTG		
ERE5F	CAGAACCTGACATTTTAAATGAAGAAATTAGGCAGGTCATGAGGAA	55	
ERE5R	TTCCTCATGACCTGCCTAATTTCTTCATTTAAAATGTCAGGTTCTG		
ERE6F	GAAGAGGTCAGGCAAATTATGAGGAAAGCCTCATTGTCCCC	55	
ERE6R	GGGGACAATGAGGCTTTCCTCATAATTTGCCTGACCTCTTC		

Table 3S: Top canonical pathways upregulated after E/F12 **(A)** and E/F24 **(B)** treatments

A

Function	Ratio	-log(p-value)	Number of molecules	Molecules
Role of IL-17A in Psoriasis	0.4620	9.16	6	S100A7,CXCL8,S100A9,CCL20,S100A8, DEFB4A/DEFB4B
Interferon Signaling	0.3820	18.10	13	IFITM3,IFIT1,IFIT3,OAS1,MX1,STAT2, IRF9,IFI35,PSMB8,STAT1,IFITM2,TAP1, IFITM1
Airway Pathology in Chronic Obstructive Pulmonary Disease	0.2500	2.68	2	CXCL8,MMP9
Pathogenesis of Multiple Sclerosis	0.2220	2.57	2	CXCL10,CCL5
Differential Regulation of Cytokine Production in Intestinal Epithelial Cells by IL-17A and IL-17F	0.1740	4.35	4	DEFB103A/DEFB103B,LCN2,CCL5, DEFB4A/DEFB4B

B

Function	Ratio	-log(p-value)	Number of molecules	Molecules
Role of IL-17A in Psoriasis	0.3850	7.19	5	S100A7,S100A9,CCL20,S100A8, DEFB4B/DEFB4B
Ascorbate Recycling (Cytosolic)	0.3330	1.58	1	GLRX
Glutathione Redox Reactions II	0.3330	1.58	1	GLRX
Interferon Signaling	0.3240	14.30	11	IFIT3,IFITM3,OAS1,MX1,IFI35,STAT2, IRF9,STAT1,TAP1,IFITM2,IFITM1
Arginine Degradation I (Arginase Pathway)	0.2500	1.45	1	ARG1
Pathogenesis of Multiple Sclerosis	0.2220	2.56	2	CXCL10,CCL5
Differential Regulation of Cytokine Production in Intestinal Epithelial Cells by IL-17A and IL-17F	0.1740	4.32	4	DEFB103A/DEFB103B,LCN2,CCL5, DEFB4A/DEFB4B
